# Association between postpartum systemic inflammation and serum calcium in healthy multiparous dairy cows: An exploratory observational analysis

**DOI:** 10.3168/jdsc.2024-0725

**Published:** 2025-04-11

**Authors:** R. Couto Serrenho, R.C. Neves, S.J. LeBlanc

**Affiliations:** 1 Department of Population Medicine, University of Guelph, Guelph, ON, Canada N1G 2W1; 2 Department of Veterinary Clinical Sciences, College of Veterinary Medicine, Purdue University, West Lafayette, IN 47907

## Abstract

Our objective was to investigate the association between serum total Ca concentration (tCa) and indicators of systemic inflammation (SI) in postpartum dairy cows. We hypothesized that the tCa-SI association varies in the first 8 d of lactation. This exploratory analysis used data from a field trial in which cows were randomly assigned to receive oral Ca supplementation after calving (OSCa), or not. Healthy multiparous cows (total n = 101; n OSCa = 51) from 2 farms in Ontario, Canada, were included. The inflammatory markers assessed were serum concentrations of albumin (a negative acute phase protein), and the positive acute phase proteins haptoglobin and serum amyloid A (SAA). Albumin, haptoglobin, and tCa were assessed at 0 (<12 h after calving), 0.5 (12 h), 1, 2, 4, 6, and 8 d after parturition; SAA was assessed at d 0, 2, and 4. Linear regression models were fitted while accounting for time as a repeated measure for each analyte, and included tCa, tCa^2^, days postpartum, parity (2 vs. ≥3), OSCa, the interactions of tCa × day postpartum, tCa^2^ × day postpartum, tCa × parity, tCa^2^ × parity, and farm as a random effect. A stepwise backward elimination approach was applied to improve model parsimony. The lowest albumin and greatest haptoglobin and SAA concentrations were observed at d 2. The association of albumin with tCa varied quadratically over time. At d 0, 0.5, 1, and 2, as tCa decreased, albumin increased, but at d 4, 6, and 8 as tCa decreased, albumin also decreased. At d 0, 0.5, and 1, when tCa <1.7 mmol/L, tCa and haptoglobin were positively associated. Total calcium <1.7 mmol/L was not observed ≥d 2. After d 1, cows with lower tCa tended to have greater haptoglobin concentrations. The greatest concentration of SAA occurred when tCa was 1.9 mmol/L. Values of tCa <1.9 mmol/L were mainly observed at d 0. At d 0, and when tCa was <1.9 mmol/L, lower tCa was associated with lesser SAA concentration. However, when tCa was >1.9 mmol/L (93% and 99% of the values observed at d 2 and 4, respectively), lower tCa was associated with greater SAA concentration. The associations between tCa and markers of SI changed in the first 8 d of lactation in clinically healthy cows. Potential interventions to modulate hypocalcemia or SI, or both, should consider their interactions. Future studies should further explore the association of the degree and duration of hypocalcemia and SI to better understand the direction and mechanisms of this relationship.

## Highlights

• Lower Ca immediately postpartum was not associated with SI.
• Lower Ca at 2, 4, 6, and 8 days postpartum pointed to greater SI.
• Time-sensitive shifts occur in the Ca-SI relationship in the first week postpartum.

## Graphical Abstract

**Figure:**
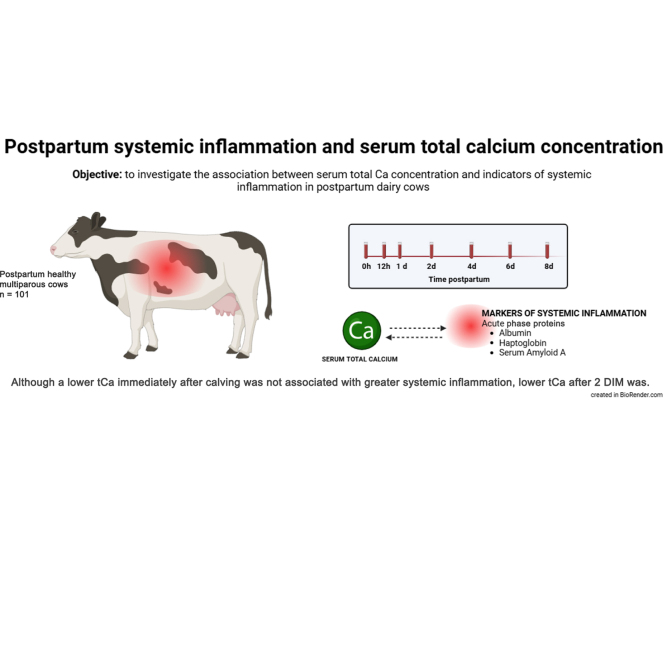
**Summary:** The degree and duration of systemic inflammation (SI) and hypocalcemia are associated with health, production, and reproductive performance, although the relationship between calcium (Ca) and SI is not fully understood. The objective of this exploratory analysis was to investigate the association between serum total Ca concentration (tCa) and indicators of postpartum SI in multiparous dairy cows. This study highlights the relationships between tCa and markers of SI change following parturition, with measurable shifts in those associations by 2 days in milk (DIM). Although lower tCa immediately after calving was not associated with SI, lower tCa after 1 DIM pointed to greater SI.

Around parturition, dairy cows inevitably experience some degree of systemic inflammation (**SI**; Bradford et al., 2015), but when excessive or dysregulated, it may contribute to inflammatory disorders (Bogado Pascottini and LeBlanc, 2020). The degree and duration of SI and hypocalcemia are associated with health, production, and reproductive performance (Bertoni et al., 2008; Neves, 2023), although the relationship between Ca and SI is not fully understood (Al-Qaisi et al., 2020; Horst et al., 2020). Albumin (**ALB**), Haptoglobin (**Hp**), and serum amyloid A (**SAA**), commonly used as inflammatory markers to assess SI in dairy cows (LeBlanc, 2023), are acute phase proteins (**APP**) produced by the liver. Upon an inflammatory insult, their concentrations change markedly in circulation over a few days (Bradford et al., 2015).

Transiently reduced blood Ca concentration in the immediate postpartum period does not seem to negatively affect milk production, but it is associated with adverse effects in multiparous cows experiencing it later in early lactation (Chapinal et al., 2011; Neves, et al., 2018a,b; Couto Serrenho et al., 2021). Assessing total calcium (**tCa**) concentration at multiple time points after calving can provide greater insight into the nature and potential consequences of changes in blood tCa in the first days postpartum (McArt and Oetzel, 2023). In multiparous cows, transient subclinical hypocalcemia (**tSCH**) appears to indicate a positive adaptation to lactation, and it is associated with better health and performance than delayed or persistent subclinical hypocalcemia (McArt and Neves, 2020; Hernandez and McArt, 2023). However, the physiological mechanisms behind these associations remain unknown (Hernandez and McArt, 2023).

Multiparous cows with blood tCa <2.2 mmol/L at 4 d postpartum exhibited lower DMI and rumination activity during the first 5 d postpartum than cows with greater tCa at that time (Seely et al., 2021; Seely and McArt, 2024). It remains unclear whether the “dyscalcemia” status is a cause or consequence of lower DMI or disease. Although a lower DMI could explain lower tCa concentration due to reduced dietary Ca absorption (Horst et al., 1994; Goff, 2000), circulating Ca deficit might also lead to a reduction in DMI (Seely et al., 2021; Neves, 2023). Excessive postpartum SI may contribute to either or both of subclinical hypocalcemia (**SCH**) and lower DMI (Kuhla, 2020; Neves, 2023). The natural decrease in tCa immediately after parturition is explained by the demand of Ca for colostrum (Goff et al., 2002). Because the best performing cows have lower tCa soon after parturition (Neves et al., 2018b; McArt and Neves, 2020), this Ca decrease may reflect advantageous metabolic adaptation to lactation (Hernandez and McArt, 2023).

Although not exclusive to the postpartum period, reduced plasma Ca concentration may be part of an adaptive response to control inflammation (Eckel and Ametaj, 2016), which could explain a changing Ca-inflammation relationship in early lactation. Our objective was to investigate the association between serum tCa and postpartum SI markers in multiparous dairy cows. We hypothesized that the relationship between inflammatory status and blood Ca concentration changes over time in the first week postpartum.

This exploratory analysis used data from a controlled trial in which cows were randomly assigned to receive oral Ca supplementation (**OSCa**; 42 g of Ca, Bovikalc, Boehringer Ingelheim Animal Health) after calving and 12 h later or not to receive Ca supplementation (Couto Serrenho et al., 2024). Healthy, second-to-fourth-lactation cows (total n = 101; OSCa n = 51) from 2 farms in Ontario, Canada, were included (herd size: farm A, n = 475; farm B, n = 460 lactating cows). Farm A milked their cows 3×/d on a double-16 parallel parlor and farm B used an automated milking system. On both farms, close-up cows were fed a neutral DCAD diet. From September to December 2021, farms were visited by the research team twice daily to enroll cows and collect blood samples. The experimental protocol for the primary study (Couto Serrenho et al., 2024) was reviewed and approved by the University of Guelph Animal Care Committee (AUP #4633).

The main outcomes of interest were serum concentrations of Hp, ALB, and SAA. Albumin, Hp, and tCa were measured at 0 (<12 h after calving), 0.5 (12 h after the first sample), 1, 2, 4, 6, and 8 d after parturition; SAA was measured at d 0, 2, and 4. Evacuated 10-mL tubes without anticoagulant (Vacutainer Precision Glide, Becton Dickinson) were used to collect the blood from the coccygeal vessels. Samples were kept on ice and processed within 2 h of collection at the University of Guelph (Guelph, ON). Tubes were centrifuged (1,500 × *g* for 15 min at ∼22°C) and 2 aliquots of serum were stored at −20°C (for tCa, ALB, Hp) or −80°C (for SAA) until laboratory analysis. At the end of the sampling period, samples were submitted to the Animal Health Laboratory, University of Guelph for serum tCa, Hp, and ALB quantification. Serum amyloid A serum samples were thawed at 22°C and assessed using a commercial ELISA kit (Multispecies SAA, Tridelta Development Ltd.). The tCa, Hp, and ALB assays' lower limits of quantification were 0.2 mmol/L, 0.1 g/dL, and 2 g/L, respectively. The interassay CV for tCa, Hp, and ALB were 0.90%, 8.96%, and 0.90%, respectively. The SAA assay lower limit of quantification was 9.40 µg/mL and the inter- and intra-assay CV were 15% and 22%, respectively.

The distributions of tCa, Hp, ALB, and SAA by day postpartum are described in Table 1. We built mixed linear regression models (PROC MIXED, SAS, version 9.4) accounting for repeated measures (day) for each marker. Full models included tCa, tCa^2^, days postpartum (0, 0.5, 1, 2, 4, 6, and 8, except for SAA: d 0, 2, and 4), parity (2 vs. 3 or 4), OSCa (yes vs. no), the interactions of tCa × day, tCa^2^ × day, tCa × parity, and tCa^2^ × parity as fixed effects, and farm as random effect. Farm was included as a random effect because we did not aim to estimate farm-specific variance but rather to improve the precision of fixed-effect estimates while accounting for between-herd variability. This approach acknowledges farm-level differences while preserving model degrees of freedom. As simulated by Gomes (2022), and tested in our dataset, in studies with sufficiently large sample sizes (n >30 per level of putative random effect), the use of random effects with a limited number of levels does not bias the coefficients of fixed effects (Gomes, 2022). Transformation of the dependent variable was applied when model residuals were not normally distributed (Hp model). A backward stepwise elimination of covariates with *P* > 0.1 was applied to the full models. We reintroduced OSCa into the final models to confirm its lack of significance (*P* > 0.3) before removing it again. Covariance structures for each model were selected based on the lowest Akaike information criterion. For the ALB model, and because of a significant tCa^2^ × day postpartum interaction, a post hoc analysis of the regressions was performed to assess differences among linear and quadratic terms across the different day postpartum (contrasts compared among slopes using the ESTIMATE statement in SAS). Graphical representations of the results from all models (ALB, Hp, and SAA) include only the range of tCa concentrations observed on each day postpartum. Because calcium metabolism in dairy cows is perceived as problematic when tCa is lower, the interpretation of the results is focused on the association of lower tCa values at specific days postpartum.

**Table 1:** Serum total Ca (tCa), haptoglobin (Hp), albumin (ALB), and serum amyloid A (SAA) concentrations in the first week of lactation (days postpartum) in clinically healthy multiparous cows (n = 101)

Variable (days postpartum)	Mean ± SD	Minimum	Q1	Median	Q3	Maximum
tCa (mmol/L)						
0^1^	2.08 ± 0.19	1.33	1.99	2.10	2.21	2.45
0.5^2^	2.05 ± 0.21	1.25	1.95	2.09	2.20	2.45
1	2.07 ± 0.21	1.39	1.97	2.11	2.21	2.42
2	2.24 ± 0.18	1.71	2.14	2.25	2.37	2.56
4	2.40 ± 0.14	1.88	2.32	2.41	2.51	2.66
6	2.43 ± 0.13	2.04	2.37	2.43	2.51	2.88
8	2.50 ± 0.13	2.09	2.44	2.50	2.57	2.83
Hp (g/L)						
0^1^	0.22 ± 0.20	0.11	0.16	0.19	0.22	1.99
0.5^2^	0.35 ± 0.24	0.14	0.23	0.31	0.38	2.10
1	0.47 ± 0.29	0.17	0.32	0.39	0.55	2.39
2	0.59 ± 0.49	0.13	0.30	0.43	0.64	2.70
4	0.45 ± 0.51	0.13	0.17	0.21	0.47	2.58
6	0.35 ± 0.54	0.12	0.16	0.19	0.26	4.15
8	0.33 ± 0.70	0.12	0.14	0.17	0.20	6.65
ALB (g/L)						
0^1^	38 ± 3	8	36	38	39	44
0.5^2^	36 ± 2	7	35	37	38	41
1	36 ± 2	5	34	36	37	40
2	35 ± 2	7	34	35	37	40
4	35 ± 3	6	33	35	37	40
6	36 ± 3	7	34	36	37	42
8	36 ± 3	8	35	37	38	42
SAA (μg/mL)						
0^1^	89 ± 57	9	39	82	138	187
2	124 ± 48	42	83	122	173	187
4	91 ± 56	9	45	87	136	188

1 Sample collected within 12 h after calving.

2 Sample collected 12 h after the first sample.

This exploratory analysis included 101 healthy Holstein cows (farm A: n = 31; farm B: n = 70) in lactation 2, 3, or 4. In the sample, 43 cows (43.4%) started their second lactation, and 51 cows (50.5%) received OSCa postpartum (blocked by parity). Although offered to the models, OSCa was not retained in any of the final models, and its removal changed the final coefficients of the retained variables by ≤4%. The descriptive statistics of tCa and the outcomes of interest are presented in Table 1. Dyscalcemia (tCa ≤2.20 mmol/L at 4 d postpartum; McArt and Oetzel, 2023) was observed in 8% of the cows. The greatest indications of SI were observed at d 2 when ALB was lowest and Hp and SAA concentrations were greatest (Table 1).

The association of ALB with tCa varied quadratically over time (tCa^2^ × d, *P* < 0.001; Figure 1a). At d 0, 0.5, 1, and 2, as tCa decreased, ALB increased, but at d 4, 6, and 8 as tCa decreased, ALB also decreased (Figure 1a). The linear and quadratic Ca terms at d 0, 0.5, 1, and 2 were not significantly different (*P* > 0.09). The linear and quadratic terms of d 4, 6, and 8 were not different (*P* > 0.4). However, linear and quadratic terms from d 0, 0.5, 1, and 2 were different from the linear and quadratic terms of d 4, 6, and 8 (*P* < 0.001). That is, lower values of tCa < d 2 were associated with greater ALB concentration but lower tCa after d 2 was associated with lower ALB concentration.

**Figure 1:**
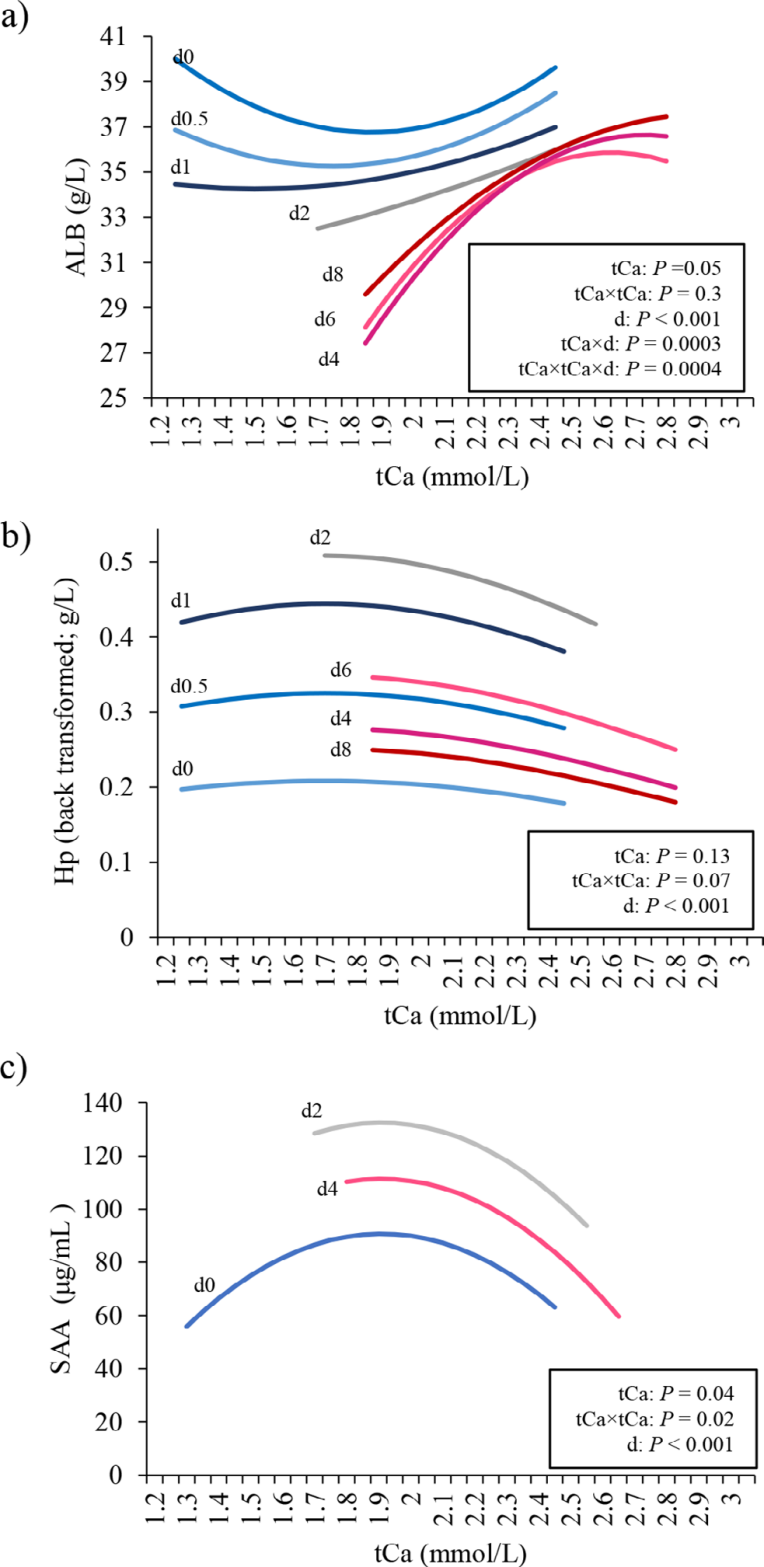
Relationships between serum concentrations of total calcium (tCa) and (a) albumin (ALB), (b) back-transformed haptoglobin (Hp), and (c) serum amyloid A (SAA) in the first week of lactation (d = DIM) in clinically healthy multiparous cows (n = 101). Representations include only tCa values observed at each day postpartum. For ALB, d 0: 66.3028 − 31.6103 tCa + 8.458 tCa^2^; d 0.5: 55.1087 − 22.727 tCa + 6.509 tCa^2^; d 1: 41.1833 − 9.189 tCa + 3.052 tCa^2^; d 2: 30.1426 − 0.8647 tCa + 1.326 tCa^2^; d 4: −52.7243 + 67.4867 tCa − 12.85 tCa^2^; d 6: −51.8626 + 64.8035 tCa − 11.86 tCa^2^; and d 8: −20.7376 + 39.7204 tCa - 6.764 tCa^2^. For Hp, d 0: exp(−2.3657 + 0.9374 tCa − 0.2754 tCa^2^); d 0.5: exp(−1.9209 + 0.9374 tCa − 0.2754 tCa^2^); d 1: exp(−1.609 + 0.9374 tCa − 0.2754 tCa^2^); d 2: exp(−1.4738 + 0.9374 tCa − 0.2754 tCa^2^); d 4: exp(−1.8519 + 0.9374 tCa − 0.2754 tCa^2^); d 6: exp(−2.0783 + 0.9374 tCa − 0.2754 tCa^2^); and d 8: exp(−2.1797 + 0.9374 tCa − 0.2754 tCa^2^). For SAA, d 0: −252.12 + 359.35 tCa − 94.1556 tCa^2^; d 2: −210.35 + 359.35 tCa − 94.1556 tCa^2^; d 4: −231.44 + 359.35 tCa − 94.1556 tCa^2^.

For Hp and SAA, the final models included tCa, tCa^2^, and day postpartum. The interpretation of these was based on the vertex point of the parabola (quadratic term). Independent of day postpartum, the greatest concentration of Hp occurred when tCa was 1.7 mmol/L (vertex value; Figure 1b). On d 0, 0.5, and 1, when tCa <1.7 mmol/L, tCa and Hp were positively associated. In other words, before d 2, a lower tCa concentration was associated with lower Hp. For example, at d 0.5, a tCa decrease from 1.6 to 1.5 mmol/L represented a decrease of 0.003 g/L (3 mg/L; 1%) in Hp. Total Ca <1.7 mmol/L was not observed ≥d 2. At d 2, 4, 6, and 8, cows with lower tCa tended to have greater Hp concentration (*P* = 0.07); for every 0.1 unit drop in tCa, Hp increased up to 4%. For example, at d 2, a tCa decrease from 2.1 to 2.0 mmol/L corresponded to an Hp increase of 0.01 g/L (10 mg/L) and a tCa decrease from 2.5 to 2.4 mmol/L corresponded to a Hp increase of 0.02 g/L (20 mg/L). The highest concentration of SAA occurred when tCa was 1.9 mmol/L, with values of 91 μg/mL on d 0, 132 μg/mL on d 2, and 111 μg/mL on d 4. Values of tCa <1.9 mmol/L were mainly observed at d 0 (Figure 1c). At d 0, if tCa was <1.9 mmol/L, lower tCa was associated with lower SAA concentration. However, when tCa was >1.9 mmol/L (most of the values observed at d 2 and 4), lower tCa was associated with greater SAA concentration (*P* = 0.02) and for every 0.1 unit drop in tCa, SAA increased up to 13%. For example, at d 2 a tCa decrease from 2.1 to 2.0 mmol/L corresponded to a SAA increase of 2.7 μg/mL, and a tCa decrease from 2.5 to 2.4 mmol/L corresponded to a SAA increase of 10.2 μg/mL.

This study explored the association between serum tCa and inflammatory markers in healthy multiparous cows in their first week of lactation. Although the positive and negative slopes as well as the mechanism of the associations between tCa concentration and markers of inflammation cannot be established, our results support changes in the relationship of tCa with SI within the first week of lactation as hypothesized. Although lower blood tCa concentrations immediately after parturition (≤24 h) were not linked with greater inflammatory status, after d 1 lower tCa concentrations were associated with greater concentrations of SI markers. Although we observed associations between tCa and SI markers, the biological significance of the magnitude of change in inflammatory markers per unit change in tCa, particularly in the Hp model, remains questionable and should be further explored in datasets that include diseased cows. Our results can help explain previous observational studies that indicate that SCH immediately after calving was not associated with impaired performance (Neves et al., 2018b) and that in multiparous cows, lower tCa at 4 d postpartum (dyscalcemia) is associated with poorer performance, independently of their tCa concentration at 1 d postpartum (McArt and Neves, 2020; McArt and Oetzel, 2023). Reduced rumination (Seely and McArt, 2024) and decreased production or reproduction associated with dyscalcemia (McArt and Neves, 2020; Seely and McArt, 2023) could be a consequence of excessive inflammation or reduced DMI, or both (Neves, 2023).

The fact that in this study lower tCa immediately after calving (≤24 h) was associated with less inflammation, as demonstrated by greater ALB and less Hp and SAA, supports the hypothesis that a transient drop in tCa immediately postpartum may be beneficial or indicative of successful adaptation to lactation (Hernandez and McArt, 2023), provided that clinical signs of hypocalcemia are absent. In a case-control study, Seminara et al. (2025) explored inflammatory markers in multiparous cows from 2 commercial farms, categorizing them by tCa status at 4 d postpartum (tCa <2.2 mmol/L) with 16 dyscalcemic cases and 32 eucalcemic controls. As in our study, Seminara et al. (2025) indicated that cows with lower tCa at 4 d postpartum had a greater acute inflammatory response. Our results support the concept that lower tCa levels beyond the immediate postpartum period may be associated with greater SI. The question remains whether the associated undesirable outcomes are a consequence of lower tCa or due to excessive inflammation (of which dyscalcemia is an indicator) that leads to reduction in DMI. Although the causal pathways and covariates that may play a role in this relationship are still to be clarified, understanding the relationship between blood tCa concentration and SI in multiparous cows during the first week postpartum is crucial to refine approaches to prevention and management of dyscalcemia.

Although some degree of SI around calving is physiologic, the concept remains poorly defined (LeBlanc, 2023). Our work included only clinically healthy cows, so with less inflammation than if diseased animals were included (Bradford et al., 2015). While excluding diseased cows may have reduced the strength of the relationship between tCa and SI, it also minimized possible confounders due to differences in management and treatments applied to sick cows. Including only healthy cows may explain the low proportion of dyscalcemic cows observed in our sample compared with previous studies (i.e., 8% vs. 30% reported by Seely et al., 2021).

This exploratory study used preexisting data, which brings limitations. Although the effect of OSCa was not significant in any of the models, a possible interaction between blood Ca concentrations and calcium supplementation on SI cannot be discounted. Additionally, differences between farms and management practices may influence these relationships, and future research should explore these aspects in more depth. Moreover, because the ratio of ionized calcium (**iCa**) to tCa in early lactation is variable and iCa is the bioactive form, studies measuring iCa to assess the relationship between calcium and SI are warranted.

This study highlights the changing relationships between tCa and markers of SI in the days following parturition. Potential interventions to modulate hypocalcemia or SI should consider their interactions. Future studies should further explore the associations of the degree and duration of hypocalcemia and SI to better understand the direction and mechanisms of this relationship and explore developing means to mitigate the proportion of cows with low tCa after d 1 and SI in early lactation.
